# Bmal1 regulates inflammatory responses in macrophages by modulating enhancer RNA transcription

**DOI:** 10.1038/s41598-017-07100-3

**Published:** 2017-08-01

**Authors:** Yumiko Oishi, Shinichiro Hayashi, Takayuki Isagawa, Motohiko Oshima, Atsushi Iwama, Shigeki Shimba, Hitoshi Okamura, Ichiro Manabe

**Affiliations:** 1 Department of Cellular and Molecular Medicine, Medical Research Institute, Tokyo Medical and Dental University, Tokyo, Japan; 2 Department of Cardiovascular Medicine, Nagasaki University Graduate School of Biomedical Sciences, Nagasaki, Japan; 3 Department of Cellular and Molecular Medicine, Graduate School of Medicine, Chiba University, Chiba, Japan; 4 Department of Health Science, School of Pharmacology, Nihon University, Tokyo, Japan; 5 Department of System Biology, Graduate School of Pharmaceutical Sciences, Kyoto University, Kyoto, Japan; 6 Department of Disease Biology and Molecular Medicine, Graduate School of Medicine, Chiba University, Chiba, Japan

## Abstract

Bmal1 (encoded by *Arntl* gene) is a core circadian clock gene that regulates various genes involved in circadian rhythm. Although Bmal1 is expressed rhythmically in macrophages, the role of Bmal1 in the regulation of their cellular function remains insufficiently understood. Here, we report that Bmal1 regulates time-dependent inflammatory responses following Toll-like receptor 4 (TLR4) activation by modulating enhancer activity. Global transcriptome analysis indicated that deletion of *Arntl* perturbed the time-dependent inflammatory responses elicited by TLR4 activation by Kdo2-lipid A (KLA). Although the recruitment of NF-κB p65 was unaffected, the acetylation status of lysine 27 of histone 3, which correlates positively with enhancer activity, was globally increased at PU.1-containing enhancers in *Arntl* ^−/−^ macrophages as compared to wild-type cells. Expression of *Nr1d1* and *Nr1d2*, encoding RevErb transcription factors, which repress enhancer RNA expression, was significantly decreased in *Arntl* ^−/−^ macrophages. Moreover, the level of H3K27 acetylation was increased by *Arntl* deletion at RevErb-dependent eRNA-expressing enhancers. These results suggest that Bmal1 controls KLA-responsive enhancers, in part by regulating RevErb-directed eRNA transcription. Taken together, the results of this study show that the clock transcription factor network containing Bmal1 controls the inflammatory responses of macrophages by regulating the epigenetic states of enhancers.

## Introduction

The circadian clock is an endogenous oscillator that drives the diurnal rhythms of physiology and behavior. In mammals, the circadian system has a hierarchical architecture, composed of a light responsive central clock in the suprachiasmatic nuclei and a peripheral clock present in all the cells of the body. The circadian clockwork is driven by a molecular feedback loop in which a heterodimer composed of circadian locomotor output cycles kaput (Clock) and brain and muscle Arnt like protein-1 (Bmal1) drives expression of two inhibitors, cryptochrome (Cry) and period (Per)^1, 2^. These feedback loops generate circadian transcription and translation rhythms. In an additional regulatory loop, Clock and Bmal1 transactivate the nuclear receptor genes *Rora*, encoding RORα, and *Nr1d1*/*2*, encoding RevErb-α and -β. RORα in turn activates and RevErbs repress transcription of *Arntl*, encoding Bmal1. In this way, these nuclear receptors function as a stabilizing loop^3^. Together, these feedback loops generate a repeated transcriptional/translational oscillator with a 24-hour period.

Recent evidence suggests that clock genes are involved in the regulation of numerous physiological process, including metabolism, development and aging. For instance, systemic deletion of *Arntl* in mice resulted in impaired circadian behavior, loss of the rhythmicity of target gene expression, acceleration of aging processes^4^, and metabolic disorders^5–7^. Immune responses also show day-night difference^8^. For instance, susceptibility to LPS-induced endotoxin shock shows diurnal variability in mice^9^. Induction of several proinflammatory cytokines was greater when mice were challenged with LPS at ZT12 (start of dark phase) than at ZT0 (start of light phase)^10^. Moreover, perturbation or desynchronization of the clock by modulating light input alters immune function^9, 11^. Because clock genes oscillate in isolated immune cells, including macrophages^12^, the peripheral cellular clock within immune cells are likely to be involved in controlling the daily function of immune cells in addition to the central oscillator in the brain.

Macrophages are an essential component of the innate immune system. Acting via various pattern recognition receptors, such as Toll-like receptors (TLRs), macrophages respond to danger signals, including pathogen-associated molecular patterns such as LPS and damage-associated molecular patterns such as HMGB1 and DNA, which are endogenous molecules associated with cell and tissue injury. For instance, TLR4 senses LPS and activates NF-κB, AP-1 and IRF3 transcription factors, triggering transcriptional events that culminate in proinflammatory activation of macrophages^13^. The peripheral clock is present in myeloid lineage cells, including macrophages. Myeloid cell-specific deletion of *Arntl* disrupted the rhythmic cycling of blood Ly6C^hi^ monocytes and predisposed mice to the development of pathologies such as acute inflammation induced by *L. monocytogenes* infection and metabolic disorders associated with high-fat diet-induced obesity^14^. In that study, Bmal1 was found to directly suppress transcription of the proinflammatory genes *Ccl2*, *Ccl8* and *S100a8*. Macrophages with low Bmal1 levels (e.g., ZT12 or circadian desynchronization) also showed higher expression of several proinflammatory cytokines^11, 15^. Based on these results it was suggested that Bmal1 acts as an anti-inflammatory molecule in monocytes/macrophages^8^. However, Bmal1’s function appears not to be limited to suppression of inflammatory activation of monocytes/macrophages. For instance, Bmal1 reportedly controls expression of *Tlr9* in macrophages, and in a TLR9-dependent mouse sepsis model, sepsis was worse in mice that underwent the model at ZT19, when *Tlr9* expression is normally at its peak^16^.

While the circadian clock greatly affects immune responses, inflammation appears to affect the clock in reverse. Wang *et al*. showed that LPS induces a phase shift in the Per2 rhythm in synchronized peritoneal macrophages^17^. LPS also transiently reduces *Arntl* mRNA levels^17, 18^. Collectively, these results demonstrate that the circadian clock and immune system are tightly linked to one another. However, the impact of the linkage between the cellular clock and the regulatory machinery of inflammatory responses and global gene regulation in macrophages is still insufficiently understood. In the present study, we analyzed the effects of Bmal1 on the transcriptome and enhancer landscape in macrophages.

## Results

### Time-dependent inflammatory responses are disturbed in *Arntl*^−/−^ macrophages

**Figure 1:**
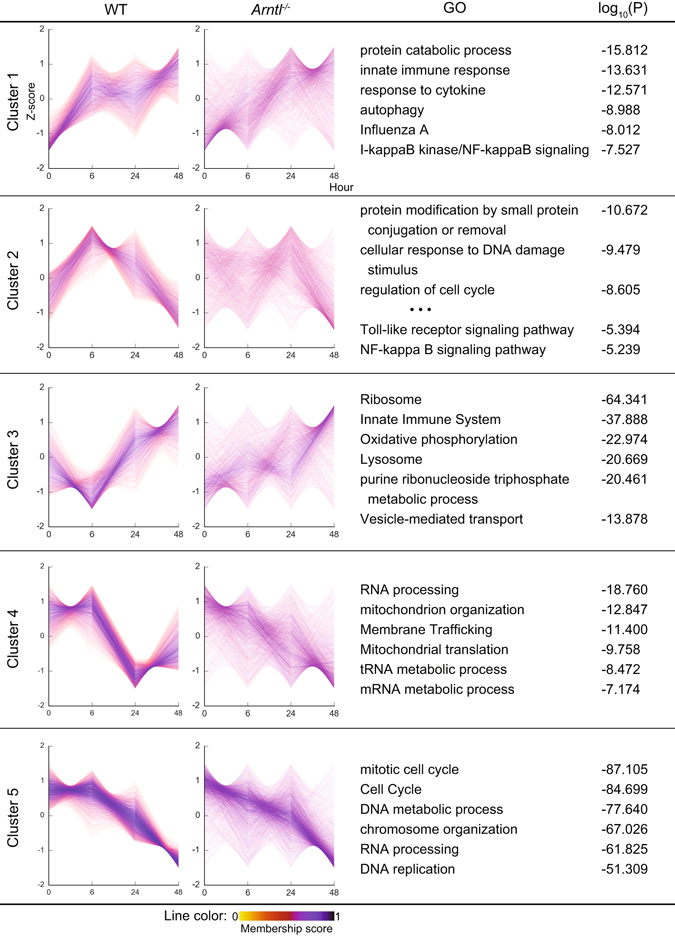
Clustering and time-course expression of genes after TLR4 activation. Bone marrow-derived macrophages prepared from WT and *Arntl* ^−/−^ mice were treated with KLA for 0, 6, 24 or 48 h, after which their transcriptomes were analyzed using RNA-seq. RefSeq genes with normalized counts ≥100 at one or more time point in WT macrophages were clustered into 5 groups using the Mfuzz clustering method^20^. Prior to Mfuzz clustering, normalized counts were standardized so that the average counts for each gene was zero and the standard deviation was one. The genes with membership scores ≥0.3 were chosen as the members of each cluster in the following analyses. Expression levels of each gene (Z-score) are shown as line graphs. The color gradient of lines from purple to pink indicates the membership score. Normalized counts of each gene in *Arntl* ^−/−^ cells were also standardized and shown as line graphs. GO terms associated with each cluster are shown.

To elucidate the genome-wide function of the clock protein Bmal1 in the machinery regulating the inflammatory responses of macrophages, we first examined the effects of *Arntl* deletion on the transcriptome. Bone marrow-derived macrophages (BMDMs) were prepared from *Arntl* ^−/−^ mice or their wild-type (WT) littermate controls. The cells were stimulated with Kdo2 lipid A (KLA), a chemically defined substructure of bacterial lipopolysaccharide (LPS) that is specifically recognized by TLR4^19^, after which the transcriptome was analyzed over the course of the inflammatory response through RNA-sequencing (RNA-seq). RefSeq genes with normalized counts ≥100 at one or more time points in WT macrophages (a total of 9,720 genes) were clustered into 5 groups using the Mfuzz clustering method^20^, and the temporal changes in gene expression in each cluster were compared between WT and *Arntl* ^−/−^ macrophages (Fig. 1). In all clusters, expression of many genes was affected by *Arntl* deletion, and expression patterns appeared to be desynchronized, particularly in clusters 1–3. Notably, the expression patterns of the KLA-induced genes in clusters 1 and 2 were strikingly disturbed in *Arntl* ^−/−^ macrophages. In WT cells, genes in clusters 1 and 2 were upregulated after KLA treatment, and the expression of core genes involved in those clusters reached a peak at 6 h. In *Arntl* ^−/−^ cells, by contrast, the clear induction pattern of those genes seen 6 h after KLA treatment was lost. Instead, expression of many of those genes reached peaks 24 h after KLA treatment, indicating a marked delay in the KLA response. Moreover, whereas expression of genes in cluster 3 sharply dipped after 6 h in WT cells, this nadir was absent in *Arntl* ^−/−^ macrophages. Gene ontology analysis indicated that genes related to inflammatory responses and cellular metabolism were significantly enriched in clusters 1–3. In particular, the GO terms “innate immune response” and “IκB kinase/NF-κB signaling” were enriched in cluster 1 (Fig. 1). Similarly, the GO terms “NF-κB signaling pathway” and “innate immune system” were enriched in cluster 2. Cluster 3 was also associated with innate immune responses. These findings suggest that the innate immune responses mediated via NF-κB signaling were disturbed in *Arntl* ^−/−^ macrophages, due to the delayed and/or desynchronized transcriptome response to TLR4 activation.

Expression of genes belonging to clusters 4 and 5 were also disturbed by *Arntl* deletion; however, the trends in the gene expression of the core members appeared to be less affected by *Arntl* deletion than in clusters 1–3. In addition, clusters 1–3 contained genes regulated by NF-κB signaling. Given these results, we will focus on the inflammatory responsive genes under the control of TLR4 signaling in the following analyses.

### Inflammatory response-related gene expression is disturbed in *Arntl*^−/−^ macrophages

The results of our RNA-seq clearly indicate that TLR4-responsive transcriptome activation is delayed or desynchronized in *Arntl* ^−/−^ macrophages. Based on these results, and given the role of Bmal1 as a central transcription factor within the cellular clock, we hypothesized that Bmal1 contributes to the time-dependent regulation of inflammation-related gene expression in macrophages. To test that idea, we first assessed the effect of TLR4 activation by KLA on the expression of clock genes. Expression of *Arnt1* and *Per1*/*2* was transiently downregulated 4 h after KLA treatment (Supplementary Fig. S1). *Cry1*/*2* was upregulated after KLA treatment; its expression showed a peak at 8 h. The effects of TLR4 activation on *Arntl* expression are consistent with earlier reports^17, 18^ and demonstrate that TLR4 activation by KLA induces time-dependent changes in clock gene expression.

**Figure 2:**
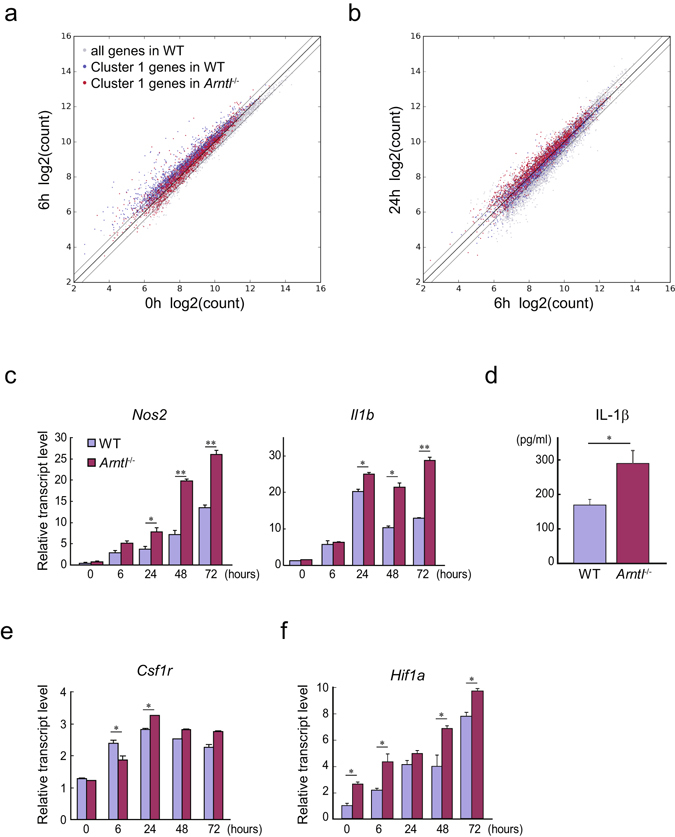
Prolonged inflammatory response in *Arntl* ^−/−^ macrophages. (**a**,**b**) Scatter plots depicting the relationship between log2 normalized counts of cluster 1 genes in WT and *Arntl* ^−/−^ macrophages treated with KLA for 0 or 6 h (**a**) and 6 or 24 h (**b**). Gray dots indicate all RefSeq genes with counts ≥100 at one or more time points in WT macrophages. Blue and red dots represent the genes belonging to cluster 1 in WT and *Arntl* ^−/−^ macrophages, respectively. Lines indicating a 1.5-fold change are shown. (**c**) Relative mRNA expression of *Nos2* and *Il1b* in WT and *Arntl* ^−/−^ macrophages treated with KLA for the indicated times. n = 3 for each group at each time point. (**d**) Levels of IL-1β protein in the culture media of WT and *Arntl* ^−/−^ BMDMs stimulated with KLA plus ATP. n = 10 for each group. (**e**,**f**) Relative mRNA expression of *Csf1r* (**e**) and *Hif1a* (**f**) in WT and *Arntl* ^−/−^ macrophages treated with KLA for the indicated times. n = 3 for each group at each time point. For qPCR in (**c**,**e** and **f**) expression levels were first normalized to those of 18 s rRNA and then further normalized to the levels in WT cells at 0 h. *p < 0.05, **p < 0.01 between WT and *Arntl* ^−/−^ cells at the same time point.

To characterize the effect of *Arntl* deletion on induction of inflammatory response-related genes, we further investigated the genes classified as cluster 1 (1,758 genes). The temporal expression profiles of these genes were evaluated by comparing the expression levels of individual genes at 0, 6 and 24 h after KLA treatment in WT and *Arntl* ^−/−^ macrophages (Fig. 2a and b, Supplementary Fig. S2). While expression of the majority of cluster 1 genes was higher at 6 h than at baseline (0 h) in WT cells, expression of these genes remained unchanged in *Arntl* ^−/−^ cells (Fig. 2a). Many cluster 1 genes in WT cells were slightly inhibited or unchanged at 24 h, as compared to their expression levels at 6 h. In *Arntl* ^−/−^ cells, by contrast, expression levels of the majority of these genes were increased at 24 h as compared with those at 6 h (Fig. 2b). These changes were also observed by comparing gene expression levels in WT and *Arntl* ^−/−^ at each time point. Expression of the majority of cluster 1 genes was higher in *Arntl* ^−/−^ cells at 0 h, lower at 6 h, and higher at 24 h than in WT cells (Supplementary Fig. S2). Collectively, expression of cluster 1 genes in *Arntl* ^−/−^ cells showed a trend toward basal activation and delayed and/or enhanced expression later, at 24 h.

Quantitative PCR (qPCR) analysis independently confirmed that expression of proinflammatory genes belonging to cluster 1, including *Nos2* and *Il1b*, remained significantly elevated 24, 48 and 72 h after TLR4 activation in *Arntl* ^−/−^ cells as compared to WT cells (Fig. 2c). Thus activation of these genes in *Arntl* ^−/−^ macrophages was both enhanced and prolonged. Consistent with the enhanced *Il1b* mRNA expression, levels of secreted IL-1β protein were significantly increased in the *Arntl* ^−/−^ BMDMs as compared to WT cells (Fig. 2d).

As in cluster 1, the upregulation of core members of cluster 2 genes seen at 6 h in WT cells was absent in *Arntl* ^−/−^ cells (Fig. 1). For instance, our qPCR showed that in *Arntl* ^−/−^ cells, expression of *Csf1r* remained low 6 h after LPS treatment but was higher after 24 h, suggesting induction of *Csf1r* was delayed and sustained in *Arntl* ^−/−^ cells (Fig. 2d). qPCR also showed that expression of *Hif1a*, belonging to the cluster 3, was enhanced at most time points in *Arntl* ^−/−^ cells. Collectively, these findings indicate that TLR4-mediated expression of a number of proinflammatory genes is enhanced and/or sustained in *Arntl* ^−/−^ macrophages.

### Bmal1 binds to PU.1-associated regulatory regions

**Figure 3:**
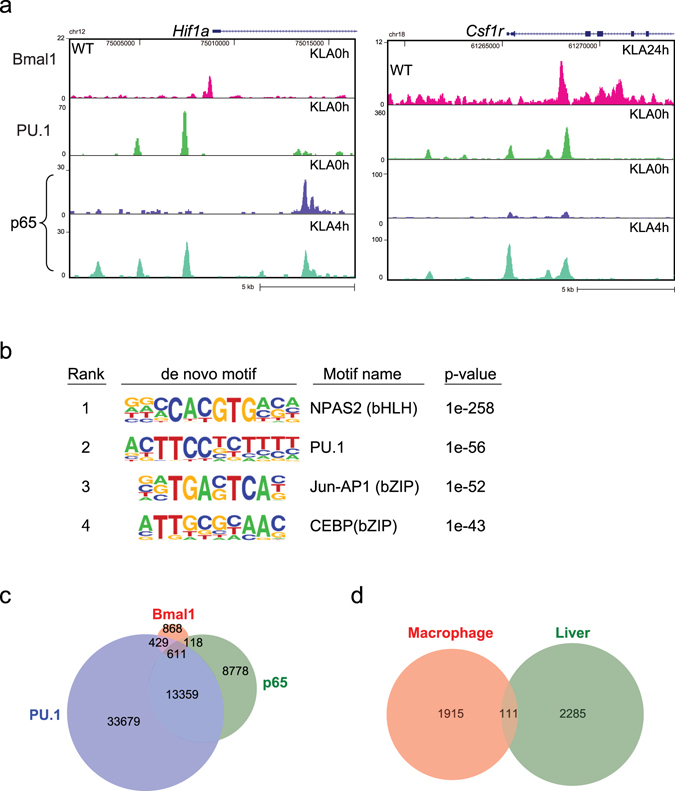
Bmal1 co-localizes with PU.1 and NF-κB. (**a**) UCSC genome browser image illustrating normalized tag counts for ChIP-seqs of Bmal1, PU.1, and p65 around the *Hif1a* and *Csf1r* loci. (**b**) *de novo* motifs identified in regions bound by Bmal1 in macrophages. (**c**) Venn diagram showing the overlap between the PU.1, p65 and Bmal1 peaks identified using ChIP-seq. Note more than half of the Bmal1 peaks (1,158 of 2,026 peaks) were also bound by PU.1 and/or NF-κB p65. (**d**) Venn diagram showing the overlap between Bmal1 peaks in macrophages and liver.

We next analyzed the mechanism by which Bmal1 affects the temporal proinflammatory gene response to TLR4 activation. To that end, ChIP-seq of Bmal1 was performed in mouse primary macrophages (thioglycolate-elicited peritoneal macrophages). As expected, Bmal1 binding to clock gene loci was observed (data not shown). Interestingly, Bmal1 binding was also observed in the regulatory regions of TLR4-inducible genes exemplified by *Hif1a* (cluster 1) and *Csf1r* (cluster 2) (Fig. 3a). In addition, *de novo* motif analysis identified a motif for basic helix-loop-helix (bHLH) transcription factors, including NPAS2, as the most highly enriched sequence followed by motifs for PU.1, a macrophage lineage-determining transcription factor, and AP-1 and C/EBP, two transcription factors known to be involved in macrophage activation (Fig. 3b). This suggests Bmal1 associates with enhancers bound by transcription factors important for the regulation of macrophage function, including PU.1, AP-1 and C/EBP.

Previous studies demonstrated that PU.1 is necessary for establishing macrophage-specific cistromes for signal-responsive transcription factors, including NF-κB^21, 22^. We found that deletion of *Arntl* affected expression of NF-κB-regulated genes (Fig. 1). Based on those observations, we hypothesized that Bmal1 may co-bind to its cognitive sites along with NF-κB and/or PU.1. Indeed, approximately half of the 2,026 Bmal1 peaks were co-occupied by PU.1 (1,040 sites; 51%). Moreover, 729 sites (36%) were co-occupied by p65, and 611 sites (30%) were co-occupied by both p65 and PU.1 (Fig. 3c). These results suggest that Bmal1 may directly affect PU.1-containing enhancers after TLR4 activation. Given that PU.1 acts as a macrophage lineage-determining transcription factor that is essential for establishment of macrophage-specific enhancers^13, 23^, the extensive co-binding of Bmal1 with PU.1 suggests Bmal1 is involved in macrophage-specific regulatory programs, such as inflammatory responses. Consistent with that idea, only a small fraction (5.5%) of macrophage Bmal1 binding peaks were shared between macrophages and the liver^24^ (Fig. 3d), suggesting the targets regulated by Bmal1 differ between macrophages and the liver. In macrophages, moreover, the target genes containing Bmal1 binding sites predicted by GREAT (genomic regions enrichment of annotations tool)^25^ were significantly associated with different GO terms than liver, with the exception of circadian rhythm-related terms (Supplementary Fig. S3). It thus appears that, aside circadian regulation, Bmal1 has different regulatory functions in macrophages than in the liver.

### Loss of *Arntl* alters the enhancer landscape

**Figure 4:**
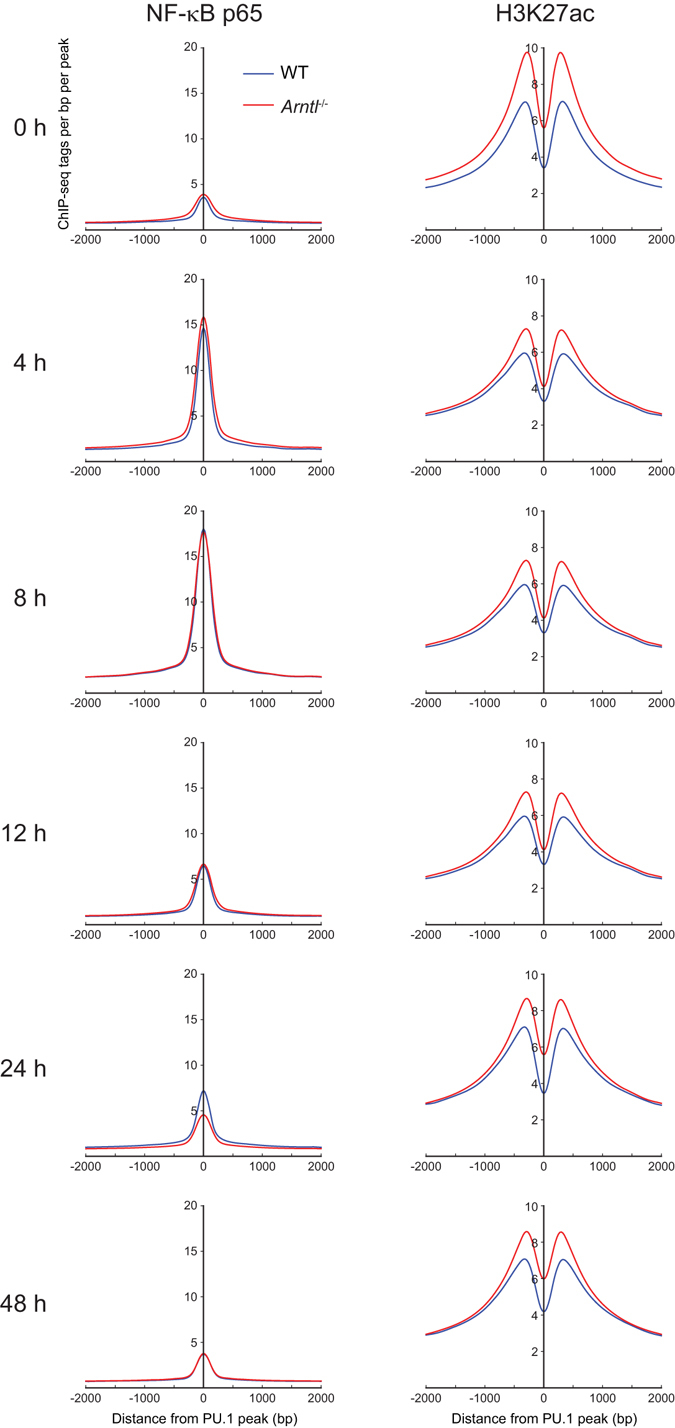
Average recruitment of NF-κB p65 to all PU.1 binding sites are unaffected by *Arntl* deletion. NF-κB p65 and H3K27ac read density in the vicinity of PU.1-dinging sites in WT and *Arntl* ^−/−^ macrophages treated with KLA for indicated times.

Given the role of NF-κB as a primary driver of TLR4-mediated inflammatory transcriptional activation, we hypothesized that perturbed NF-κB binding, namely delayed and prolonged association of NF-κB in response to TLR4 activation, is the mechanism responsible for the altered temporal proinflammatory gene expression in *Arntl* ^−/−^ macrophages. To test that idea, we performed ChIP-seq of the p65 component of NF-κB in WT and *Arntl* ^−/−^ macrophages, after which the temporal pattern of p65 binding was analyzed in WT and *Arntl* ^−/−^ macrophages. Consistent with previous studies^21, 26^, the average p65 binding was increased at PU.1-binding sites 4 h and 8 h after KLA treatment in WT macrophages, and was then lower at 12 h and later (Fig. 4, left column). Unexpectedly, however, *Arntl* deletion did not affect average p65 association with PU.1 loci (Fig. 4, left column).

The unexpected finding that p65 recruitment was unaffected by loss of *Arntl* prompted us to analyze the enhancer landscape. To assess global enhancer activity, we performed ChIP-seq of lysine 27-acetylated histone H3 (H3K27ac), which is positively associated with transcriptional activity^27, 28^. Interestingly, average H3K27 acetylation levels at all PU.1 binding sites were higher in *Arntl* ^−/−^ than WT macrophages at baseline and throughout the course of the response after KLA stimulation (Fig. 4, right column).

**Figure 5:**
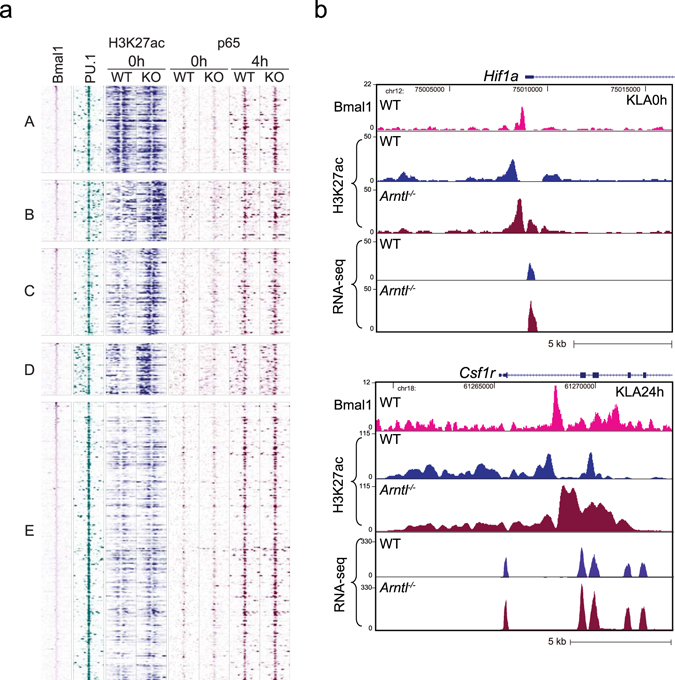
Loss of Bmal1 enhances H3K27 acetylation at Bmal1 binding sites. (**a**) Heatmap for Bmal1, PU.1 and p65 binding and H3K27 acetylation within 2 kb around the center of the top 500 Bmal1-PU1 co-binding sites in untreated cells. ChIP-seq tags for Bmal1, PU.1 and H3K27ac at baseline, were grouped into 5 clusters using the K-means clustering algorism. Corresponding p65 ChIP-seq tag densities in cells at baseline (0 h) and 4 h after KLA treatment are also shown. (**b**) Genome browser snapshots showing Bmal1 and H3K27ac ChIP-seq and RNA-seq signals at *Hif1a* and *Csf1* loci.

To further assess epigenetic changes, we first analyzed the density of H3K27ac in PU.1/Bmal1 co-binding regions to analyze possible direct effects of the loss of Bmal1. ChIP-seq tags for Bmal1, PU.1 and H3K27ac around the top 500 Bmal1 binding sites at baseline, were grouped into 5 clusters using the K-means clustering algorism. A heat map of Bmal1, PU.1 and H3K27ac reads around PU.1 peaks shows that while H3K27ac levels in cluster A were unchanged or decreased in *Arntl* ^−/−^ cells, H3K27ac levels in clusters B, C and D were clearly higher in *Arntl* ^−/−^ cells than WT cells under basal conditions (Fig. 5a). In agreement with the results of the top 500 Bmal1 sites, average H3K27ac levels around all Bmal1-PU.1 co-binding sites were increased in *Arntl* ^−/−^ cells as compared to WT cells (Supplementary Fig. S4). The changes are exemplified by the KLA-inducible *Arntl* target genes *Hif1a* and *Csf1r* (Fig. 5b). These results indicate that loss of Bmal1 leads to increased H3K27 acetylation at most enhancers to which Bmal1 and PU.1 bind.

**Figure 6:**
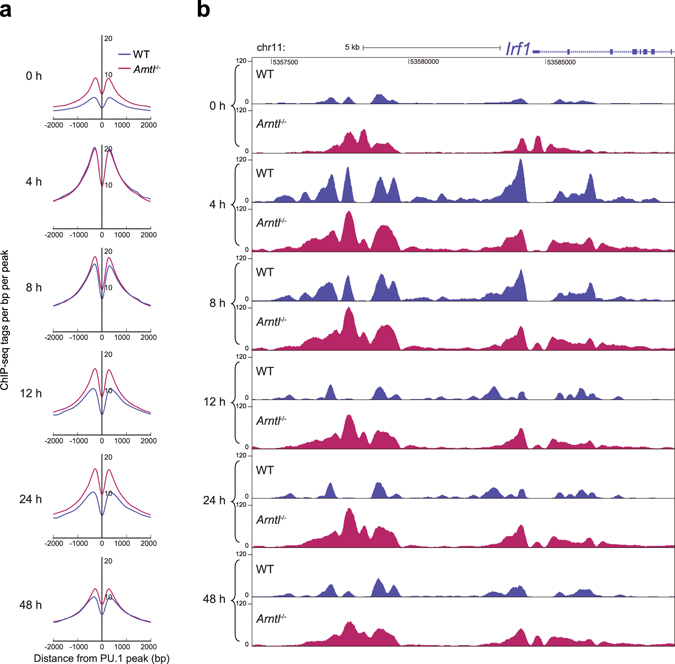
H3K27 acetylation at PU.1-bound, KLA-activated enhancers is augmented in *Arntl* ^−/−^ macrophages. (**a**) Density of H3K27ac reads at the PU.1-bound enhancers activated by KLA treatment at 4 h. (**b**) UCSC genome browser image illustrating ChIP-seq signals for H3K27ac at the *Irf1* locus in WT and *Arntl* ^−/−^ macrophages treated with KLA for the indicated times.

Although *Arntl* deletion globally affected the H3K27ac states at PU.1 sites (Fig. 4 right column), only a minor fraction of PU.1 sites were cobound by Bmal1 (Fig. 3c). Therefore, the differential deposition of H3K27 acetylation at PU.1-contining enhancers cannot be fully explained as a primary effect of the loss of Bmal1 binding. Accordingly, we further characterized the mechanism underlying the changes in H3K27 acetylation. TLR4 activation can both activate and repress PU.1-bound enhancer activity concomitantly with changes in H3K27 acetylation^26^. To dissect the epigenetic effect of the loss of Bmal1 in line with our focus on KLA-activated genes in the present study, we focused on the PU.1 enhancers at which H3K27ac was increased by KLA. Furthermore, to focus on early KLA-activated enhancers, we analyzed the PU.1 sites at which H3K27ac tags were increased ≥2-fold as compared to the steady state 4 h after KLA stimulation (5,572 peaks). In WT cells, H3K27ac levels at these KLA-activated enhancers were increased at 4 h and then gradually declined (Fig. 6a). By contrast, the average deposition of H3K27ac was already high at baseline in *Arntl* ^−/−^ macrophages. And while H3K27ac levels were comparable between the two genotypes 4 h after KLA, the elevation in H3K27ac persisted beyond 4 h in *Arntl* ^−/−^ cells. These altered H3K27ac states are exemplified by the *Irf1* locus (Fig. 6b). Moreover, the persistent activation of KLA-responsive enhancers appears to be in agreement with the delayed and prolonged mRNA response to KLA treatment for many genes in clusters 1–3 (Fig. 1). Collectively then, these findings suggest the loss of *Arntl* induces an increase in baseline enhancer activity at inflammatory genes, reduces the responsiveness to TLR4 activation, and prolongs inflammatory responses.

### Reduced NR1D1 and NR1D2 (RevErbα/β) activity modifies the enhancer activity in *Arntl*^−/−^ macrophages

**Figure 7:**
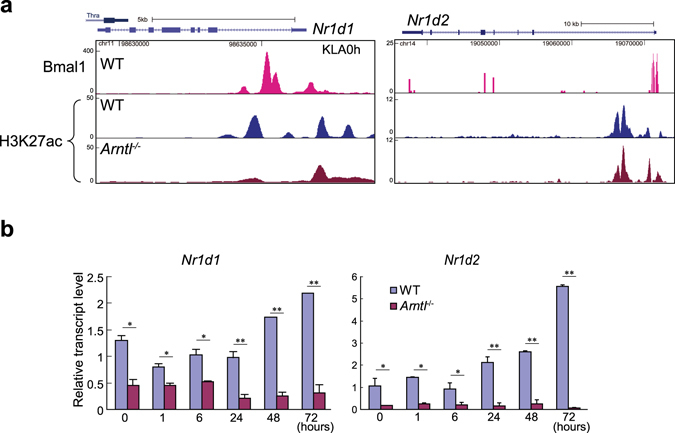
*Nr1d1* and *Nr1d2* expression is suppressed in *Arntl* ^−/−^ macrophages. (**a**) Genome browser snapshots of Bmal1 and H3K27ac ChIP-seq and RNA-seq results at *Nr1d1* and *Nr1d2* loci. (**b**) Relative expression of *Nr1d1* and *Nr1d2* mRNA in WT and *Arntl* ^−/−^ macrophages treated with KLA for indicated times. n = 3 for each group at each time point. *p < 0.05, **p < 0.01. between WT and *Arntl* ^−/−^ at the same time point.

To elucidate the mechanism by which Bmal1 modulates H3K27 acetylation globally on PU.1 sites, we hypothesized that clock-controlled transcription factors are involved. Of particular interest were RevErb-α and RevErb-β, encoded by *Nr1d1* and *Nr1d2*, respectively, because these nuclear receptors have been shown to regulate expression of genes involved in the control of metabolism and inflammatory responses in macrophages^10, 29^. In addition, *Nr1d1* and *Nr1d2* are known targets of Bmal1^30^. Consistent with their involvement, our ChIP-seq revealed several Bmal1 binding peaks in the regulatory regions of *Nr1d1* and *Nr1d2* loci (Fig. 7a), and levels of *Nr1d1* and *Nr1d2* expression were markedly reduced in *Arntl* ^−/−^ macrophages throughout the course after KLA stimulation (Fig. 7b).

**Figure 8:**
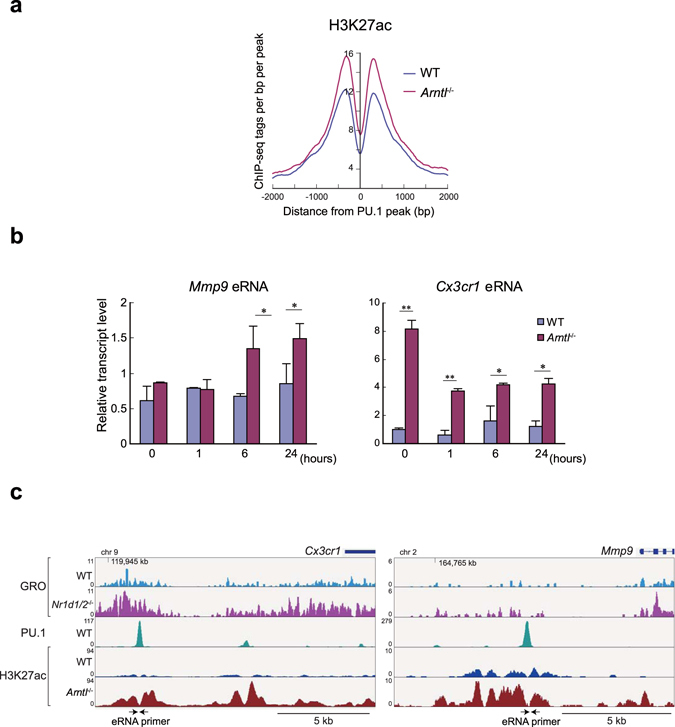
RevErb-dependent eRNA transcription was de-repressed in *Arntl* ^−/−^ macrophages. (**a**) Average H3K27ac read density around PU.1 binding sites adjacent to the RevErb-regulated eRNA-expressing enhancers in WT and *Arntl* ^−/−^ macrophages at steady state. (**b**) qPCR analysis of eRNAs transcribed at the *Mmp9* and *Cxcr1* enhancers. eRNA expression was compared between WT and *Arntl* ^−/−^ macrophages treated with KLA for indicated times. n = 3 for each group at each time point. *p < 0.05 between WT and *Arntl* ^−/−^ at the same time point. (**c**) Genome browser snapshots of GRO-seq signals in WT and *Nr1d1*/*Nr1d2* double knockout cells, and ChIP-seq signals for PU.1 and H3K27ac in WT and *Arntl* ^−/−^ cells at steady state.

RevErbs reportedly repress enhancer activity by inhibiting eRNA transcription^29^. To test whether dysregulation of eRNA transcription modulates enhancer activity in *Arntl* ^−/−^ cells, we took advantage of previously reported global run-on sequencing data (GRO-seq) from *Nr1d1*/*Nr1d2* double knockout (DKO) macrophages^29^. To identify eRNA-expressing enhancers, we first identified regions with significant nascent RNA signals determined with GRO-seq in WT or DKO cells. After signals detected at gene bodies were excluded, the nascent RNA signals with read counts increased ≥1.5-fold in *Nr1d1*/*Nr1d2* DKO cells as compared to WT cells were identified. The resultant 575 regions were likely to include the regions in which eRNA expression was upregulated by DKO. Further, we identified 330 regions with PU.1-binding among the eRNA-expressing enhancers. Interestingly, the acetylation status of H3K27 around those eRNA-expressing, PU.1-binding enhancers was markedly higher in *Arntl* ^−/−^ than WT macrophages (Fig. 8a), suggesting the reduced RevErb-mediated suppression of eRNA de-repressed the enhancers. Our qPCR results confirmed that the eRNA expression located at −5 kb in *Mmp9* and +28 kb in *Cx3cr1*, both of which are targets of RevErb-mediated repression^29^, was significantly increased in *Arntl* ^−/−^ macrophages (Fig. 8b). And H3K27ac was also increased at the PU.1-containing enhancers (Fig. 8c). Collectively, these results suggest that epigenetic dysregulation of the response to inflammatory TLR4 activation in *Arntl* ^−/−^ cells is at least partly mediated by dysregulation of eRNA transcription due to impaired RevErb function.

## Discussion

In the present study, we obtained evidence that the circadian transcription factor Bmal1 plays an essential role in the global control of inflammatory gene regulation in macrophages, thereby demonstrating that Bmal1 is an important regulator of inflammatory responses. Consistent with our findings, previous studies showed enhanced production of the proinflammatory cytokines TNF-α and IL-6 in *Anrtl* ^−/−^ macrophages^18^; in ZT12 macrophages, in which the *Arntl* levels are the lowest^15^; and in macrophages from a chronic jet lag model, in which *Arntl* expression was repressed^11^. Of particular note is that *Arntl* deletion enhanced and prolonged responses of inflammatory genes to TLR4 activation (Figs 1 and 2). As shown by the gene expression trends in clusters 1 and 2 (Fig. 1), many inflammatory genes are transiently induced early (6 h) but suppressed at later times (24 h) in WT macrophages. This suppression at 24 h was impaired by *Arntl* deletion (Fig. 1). Importantly, previous studies have shown that both the early induction and late repression of inflammatory response-related genes are actively controlled by extensive networks of signaling molecules and transcriptional and metabolic machinery^26, 31^. Notably, expression of *Arntl1* was transiently decreased at 4 h and recovered at later times (Supplementary Fig. S1). While it has been proposed that Bmal1 acts as a gatekeeper for inflammatory responses^8^, our findings suggest Bmal1 has a wider role in the time-dependent transcriptional regulation occurring in response to inflammatory stimuli in macrophages, such as late inflammatory gene repression. The global effects of Bmal1 on H3K27 acetylation at PU.1-binding enhancers (Fig. 4) also support that idea. The observation that LPS induces transient *Arntl* suppression and a phase shift in the Per2 rhythm^17, 18^ in synchronized macrophages is also consistent with the notion that the cellular clock is integral to the time-dependent regulation of gene expression after TLR4 activation.

Our ChIP-seq results show that at nearly half its binding sites, Bmal1 colocalizes with the macrophage lineage-determining transcription factor PU.1 (Fig. 3c). This is analogous to the co-binding of Bmal1 with PDX1 at enhancers in pancreatic beta cells^24^ and suggests that the targets of Bmal1 are cell type-specific. Consistent with that, most Bmal1 binding sites in beta cells are distinct from those in the liver^24, 32^. We similarly found that macrophages and the liver have only a small fraction of Bmal1 binding sites in common (Fig. 3d). Moreover, the functions of the target genes appear to differ between macrophages and the liver (Supplementary Fig. S3). Of particular interest is that ~36% of Bmal1 binding sites are also bound by NF-κB, which is the primary driver of the innate immune response (Fig. 3c). This suggests integration of Bmal1 into the immune control circuitry in macrophages. Previous studies identified multiple mechanisms interacting between Bmal1 and NF-κB via interference with CLOCK^33^, interaction with PRC2^14^, and induction of Rev-Erbα and RORα^10^. These findings all suggest Bmal1 is involved in the transcriptional regulatory mechanism governed by NF-κB. It was therefore surprising that *Arntl* deletion had no impact on the average binding of p65 to PU.1 throughout the inflammatory response after TLR4 activation. Despite the unchanged p65 binding, H3K27 acetylation at PU.1-binding enhancers was altered by *Arntl* deletion (Fig. 3), indicating global epigenetic modulation.

We observed that in *Arntl* ^−/−^ cells, H3K27 acetylation in the region around enhancers activated by TLR4 is increased at baseline and shows a blunted response at 4 h as well as prolonged activation (Fig. 6). These observations are in agreement with the changes in KLA-inducible gene expression patterns (Fig. 1). As with all PU.1 binding sites, average p65 binding at these KLA-activating enhancers was unaffected by *Arntl* deletion (data not shown), suggesting involvement of epigenetic mechanisms independent of p65 binding. A direct effect of the loss of Bmal1 binding could account for the epigenetic changes at direct Bmal1 target genes such as *Hif1a* and *Csf1r* (Fig. 5). But clearly the loss of Bmal1 had much wider effects on enhancers, even those without detectable Bmal1 binding. In addition, while the loss of Bmal1 enhanced H3K27 acetylation at many PU.1 enhancers, it also reduced H3K27 acetylation at a minor fraction of target enhancers, exemplified by those at *Nr1d1* (Fig. 7). Bmal1 thus appears to exert both activating and repressing effects at its cognitive regulatory regions. It will be important to elucidate how this dual function of Bmal1 is regulated.

Bmal1 also appears to widely exert indirect effects on PU.1 enhancers (Fig. 4). One of the indirect mechanisms identified in the present study is RevErb-mediated eRNA regulation. RevErbs function as transcriptional repressors by recruiting the NCoR-HDAC3 corepressor complex to the regulatory regions of target genes^34–36^, and by repressing eRNA transcription, which modulates enhancer function^29^. RevErb expression was suppressed in *Arntl* ^−/−^ macrophages (Fig. 7), which is consistent with the current cellular clock model in that RevErbs are directly regulated by Bmal1^37^. In *Arntl* ^−/−^ macrophages, eRNA expression in a subset of RevErb target genes, including *Mmp9* and *Cx3cr1*, were de-repressed (Fig. 8). More importantly, our results indicate that the loss of Bmal1 increases H3K27 acetylation at enhancers where eRNA transcription is repressed in a RevErb-dependent manner^29^. This suggests Bmal1 regulates enhancer activity in part through RevErb-dependent eRNA repression. In the present study, we were able to identify a small number of eRNA-expressing enhancers to which PU.1 bound. Because levels of eRNA expression are generally lower than levels of mRNA expression, our identification of eRNA-expressing regions may not be sufficiently sensitive to identify eRNA-expressing enhancers. It is therefore possible that eRNA-mediated changes in enhancer activity contribute much more broadly to the epigenetic effects of the loss of Bmal1 than we were able to detect.

Our results suggest Bmal1 controls the timing of gene expression in response to inflammatory activation by regulating the epigenetic status of enhancers, in part through regulation of RevErbs and eRNA transcription in macrophages. These findings provide insight into the time-dependent control of macrophage inflammatory responses. Intricate and coordinated mechanisms affecting transcription, the epigenome and various signaling and metabolic pathways control the initial proinflammatory response and the later dampening of proinflammatory function and activation of pro-resolution function in macrophages^26, 38^. The machinery of the cellular clock, including Bmal1 and RevErbs, appears to be integral to this time-dependent regulation of macrophage function, which is essential for proper activation and resolution of inflammation. Imbalance in the regulation of inflammatory activation and resolution can lead to chronic inflammation, which is a pivotal contributor to the development of chronic non-communicable diseases (NCDs) such as cardiovascular and metabolic diseases^26^. In that regard, it is noteworthy that deficiencies in clock genes result in cardiovascular and metabolic disease phenotypes in mice^39, 40^. Given that shift work is known to increase the risk for cardiovascular and metabolic diseases and associates with increased inflammatory markers^41, 42^, it will be important to test whether disturbance of clock-dependent regulation of inflammatory time underlies chronic inflammation in NCDs. In particular, our results demonstrate that Bmal1 is important not only as a gatekeeper for inflammatory activation in macrophages^8^, but also for the later damping of inflammatory gene expression. Chronic inflammation in NCDs is characterized by failed resolution of inflammation^43^. Future studies will need to address whether Bmal1 dysfunction is involved in promoting chronic inflammation via its multiple regulatory functions that contribute to inflammatory activation and resolution.

### Experimental Procedure

#### Reagents

Kdo2-lipid A was purchased from Avanti Polar Lipids and processed as described (http://www.lipidmaps.org/protocols/).

### Animals

*Arntl* ^−/−^ mice were generated on a C57BL/6 background as described previously^39^. Mouse thioglycollate-elicited macrophages and bone marrow-derived macrophages were isolated from male 6- to 9-week-old C57BL/6J (Charles River laboratories) and *Arntl* ^−/−^ mice. All animal experiments were approved by the Experimental Animal Care and Use Committee of Tokyo Medical and Dental University and strictly adhered to the guidelines for animal experiments of the institute.

### Cell Culture

We used BMDMs for all experiments except ChIP-seq for Bmal1. For culture of BMDMs, bone marrow was collected from mice by perfusing the medullary cavities of femurs, tibias and iliac bones, after which the bone marrow cells were cultured for 6 days in RPMI-1640 medium containing 10% FCS and 20 μg/ml M-CSF (R&D). Peritoneal macrophages were harvested by lavage 3 days after intraperitoneal injection of 3 ml of 3% thioglycollate medium (http://www.lipidmaps.org/protocols/). The cells were then cultured overnight and adherence selected.

### RNA and qPCR analysis

Total RNA was isolated from cells, purified using RNeasy columns and treated with RNase-free DNase according to the manufacturer’s instructions (Qiagen). For quantitative PCR (qPCR) analysis, 1 nM of cDNA or ChIP DNA was used for real-time PCR with gene- or locus-specific primers. qPCR was performed on an Applied Biosystems StepOne Plus system using SYBR GreenER master mix (Invitrogen) and the following protocol: incubation for 10 min at 50 °C and 10 min at 95 °C followed by 40 cycles of 15 s at 95 °C and 30 s at 60 °C. Primer sequences for RT-qPCR analysis are listed in Supplementary Table S1.

### IL-1β ELISA

BMDMs were stimulated with KLA (100 ng/ml) for 4 h, followed by ATP (5 mM/ml) treatment for 16 h. Levels of IL-1β in culture media were quantified using a mouse IL-1β ELISA kit (R&D, #MLB00C) according to the manufacturer’s instructions.

### Chromatin immunoprecipitation (ChIP)

For ChIP assays, anti-NF-κB p65 (sc-372) antibody was purchased from Santa Cruz Biotechnology; anti-Bmal1 (ab93806) and anti-H3K27Ac (ab4729) antibodies were from Abcam. For Bmal1 ChIP, 2 × 10^7^ thioglycollate-elicited primary peritoneal macrophages were used. For NF-κB p65 and H3K27ac ChIP, 2 × 10^7^ bone marrow-derived macrophage were used. After crosslinking in 1% formaldehyde, cells were resuspended in buffer containing 10 mM HEPES/KOH (pH 7.9), 85 mM KCl, 1 mM EDTA, 0.5% IGEPAL CA-630 and protease inhibitors and incubated for 5 min. The cells were then spun down and resuspended in 500 μl of lysis buffer (50 mM Tris/HCl [pH 7.4], 1% SDS, 0.5% Empigen BB, 10 mM EDTA) with protease inhibitors, and the chromatin samples were sheared by sonication. The lysates were diluted with 750 μl of dilution buffer (20 mM Tris/HCl, 100 mM NaCl, 0.5% Triton X-100, 2 mM EDTA) and 1% was used as input DNA. With the remaining samples, immunoprecipitation was carried out overnight using Dynabeads protein G coated with specific antibody. The beads were then washed twice with wash buffer I (20 mM Tris/HCl, 150 mM NaCl, 0.1% SDS, 1% Triton X-100, 2 mM EDTA), twice with wash buffer II (10 mM Tris/HCl, 250 mM LiCl, 1% IGEPAL CA-630, 0.7% Na-deoxycholate, 1 mM EDTA), once with TE plus 0.2% triton X-100, and once with TE plus 50 mM NaCl. The beads were then eluted with elution buffer (TE, 2% SDS), after which the DNA crosslinking was reversed, and the DNA was purified using a PCR purification kit (Qiagen) according to the manufacturer’s instructions.

### Sequencing library preparation

ChIP-seq libraries were prepared using a NEBNext Ultra DNA library prep kit for Illumina according to the manufacturer’s instructions. RNA-seq libraries were prepared using a NEBNext Ultra RNA library prep kit for Illumina according to the manufacturer’s instructions. Libraries were PCR-amplified for 12–15 cycles, and sequenced on a GAIIx, HiSeq. 1500 or HiSeq. 2500 (Illumina).

### High-throughput sequencing and data analysis

For macrophage Bmal1, p65 and PU.1 ChIP-seq, reads were mapped to the mm9 mouse genome using STAR (Dobin *et al*., 2013). For liver, Bmal1 reads were mapped to the mm9 using bowtie^44^. Peak calling and annotation were performed using HOMER^21^. Peaks that overlapped with blacklisted regions^45^ and simple repeat regions were removed. For ChIP-seq analyses, Homer-identified peaks whose scores were ≥20 were selected as high-confidence peaks, except liver Bmal1 peaks, which were used irrespective of scores in downstream analyses. For macrophage Bmal1 ChIP-seq, Bmal1 peaks were pooled from 3 experimental conditions (cells treated with KLA for 0, 4 and 24 h). The data used for liver Bmal1 ChIP-seq analysis was from ZT8 cells, as that time point had the largest the number of identified peaks in a previous report^32^. For GRO-seq analyses, de novo transcripts were identified using Homer’s findPeaks program with the -groseq option. De novo transcripts that overlapped with blacklisted regions and simple repeat regions were removed. Gene ontology analyses were performed using Metascape software (http://metascape.org/)^46^. Motif analyses were performed using HOMER. PU.1 Publically available ChIP-seq, RevErb ChIP-seq and GRO-seq results were downloaded from GEO (GSE62826, GSE45914, and GSE39860). All RNA-seq and ChIP-seq data are available in the GEO under the accession number GSE95712.

### Statistical Analysis

Differences among more than two groups were analyzed using one-way ANOVA followed by Tukey-Kramer post-hoc tests. Values of *p* < 0.05 were considered statistically significant, except where otherwise noted. All data are shown as means ± SEM.

## Electronic supplementary material

Supplementary Information
