# Supplementary material for: Bmal1 regulates inflammatory responses in macrophages by modulating enhancer RNA transcription
Source: Sci Rep. 2017 Aug 1;7:7086. doi: 10.1038/s41598-017-07100-3 (PMC5539165; doi:10.1038/s41598-017-07100-3)
Supplement: Supplementary file 1 — Supplementary Information [file 41598_2017_7100_MOESM1_ESM.pdf]

Supplementary information

Title:

Bmal1 regulates inflammatory responses in macrophages by modulating enhancer RNA transcription

Yumiko Oishi, Shinichiro Hayashi, Takayuki Isagawa, Motohiko Oshima, Atsushi Iwama, Shigeki Shimba, Hitoshi Okamura, and Ichiro Manabe

Supplementary Figure 1 Expression of clock genes in KLA-treated macrophages

Wild-type BMDMs were treated with KLA (100μg/ml) for indicated times, and expression levels of clock genes were analyzed by qPCR. Expression levels were first normalized to those of 18s rRNA and then further normalized to the levels at 0 h

Supplementary Figure 2 Effects of *Antl* deletion on cluster 1 gene expression

Scatter plots depicting the relationship between log2 normalized counts of cluster 1 genes in WT and *Arntl*$^{-/-}$ macrophages treated with KLA for indicated time periods. Gray dots indicate all RefSeq genes with counts ≥100 at one or more time points in WT macrophages. Red dots represent the genes belonging to cluster 1.

Supplementary Figure 3 Predicted functions associated with Bmal1 peaks in macrophages and the liver

Regulatory functions of Bmal1 binding sites were analyzed using GREAT program (http://bejerano.stanford.edu/great/public/html/index.php) using the default setting. Significantly enriched gene ontologies are shown.

Supplementary Figure 4 Distribution of p65 and H3K27ac at Bmal1-PU.1 cobinding sites

Shown are the average p65 and H3K27ac read densities around PU.1 binding sites that were also bound by Bmal1. Note that the average read densities for all PU.1 binding sites are shown in Fig. 4.

Supplementary Table 1

qPCR primers

Name	Sequence
Nos2 Fw	AGCCTTGCATCCTCATTGG
Nos2 Rv	CACTCTCTTGCGGACCATCT
Il1b Fw	TGGGCCTCAAAGGAAAGAAT
Il1b Rv	CAGGCTTGTGCTCTGCTTGT
Nr1d1 Fw	ACGACCCTGGACTCCAATAA
Nr1d1 Rv	CCATTGGAGCTGTCACTGTAGA
Nr1d2 Fw	ACAGAAATAGTTACCTGTGCAACACT
Nr1d2 Rv	GACTTGCTCATAGGACACACCA
Csf1r Fw	AAGCAAGATCTGGACAAAGAGG
Csf1r Rv	ACGTTTCGAGCTGCTACGTC
Hif1a Fw	GCACTAGACAAAGTTCACCTGAGA
Hif1a Rv	CGCTATCCACATCAAAGCAA
18s Fw	GCAATTATTCCCCATGAACG
18s Rv	GGGACTTAATCAACGCAAGC
Mmp9 eRNA Fw	AAGATGGGGGAAATGGTAGG
Mmp9 eRNA Rv	ACTTGGCAGGCAGAGTGAGT
Cx3cr1 eRNA Fw	CTGCCTCAGGGAGAAACAAG
Cx3cr1 eRNA Rv	CTGCAACTCTCAGCAACCAG

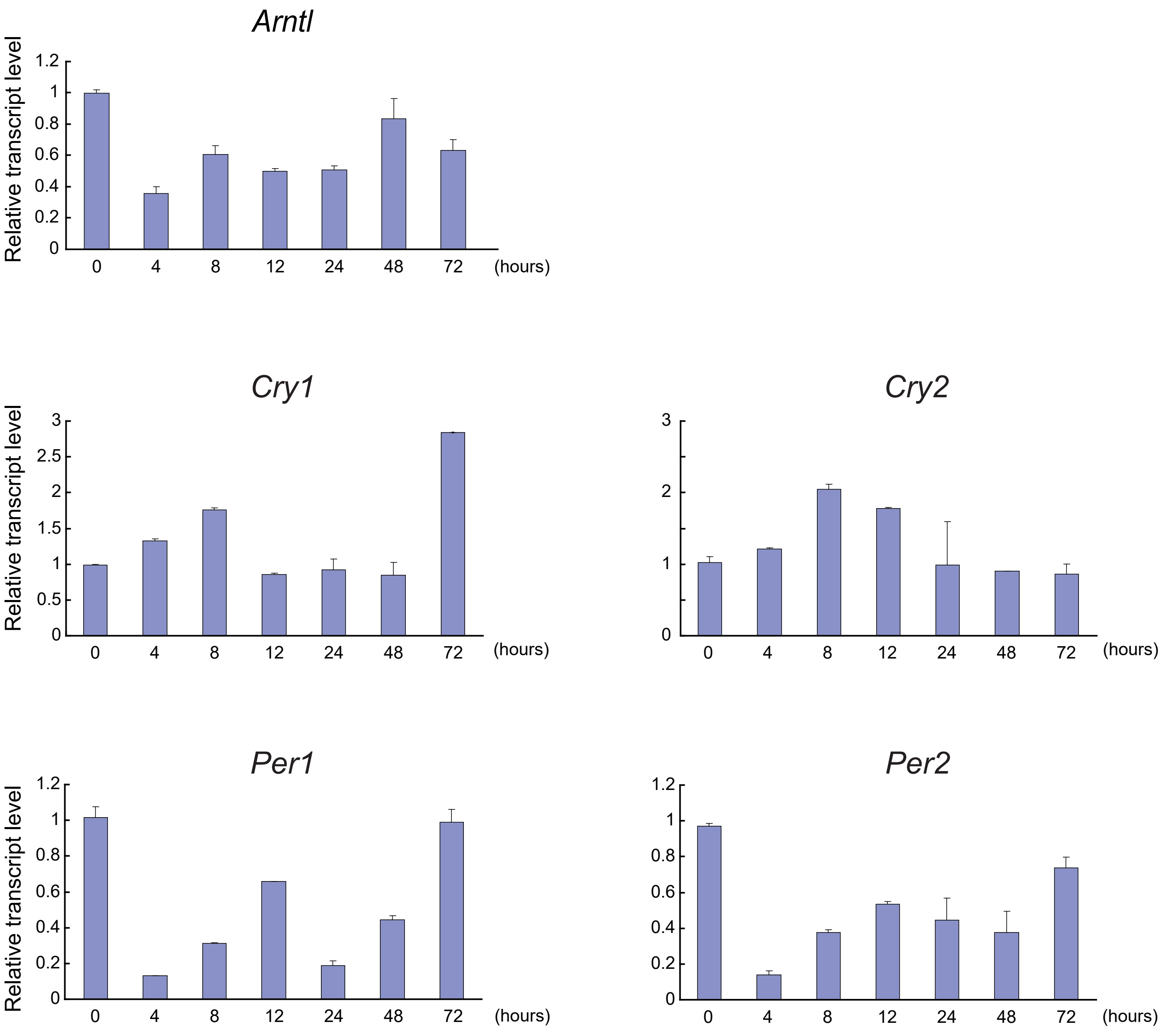

Oishi et al. Supplemenatary Figure S1

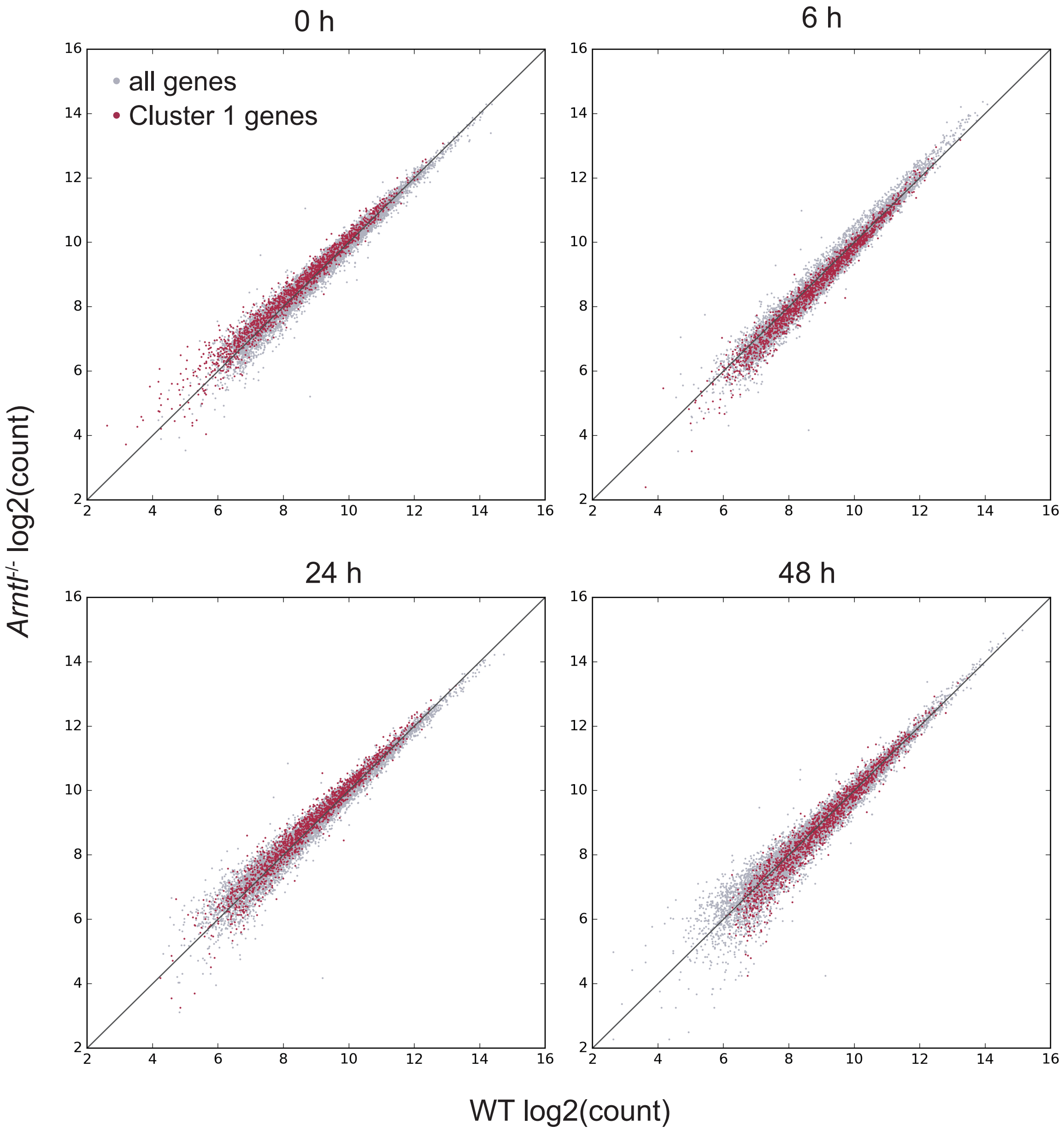

Oishi et al. Supplemenatary Figure S2

Macrophage

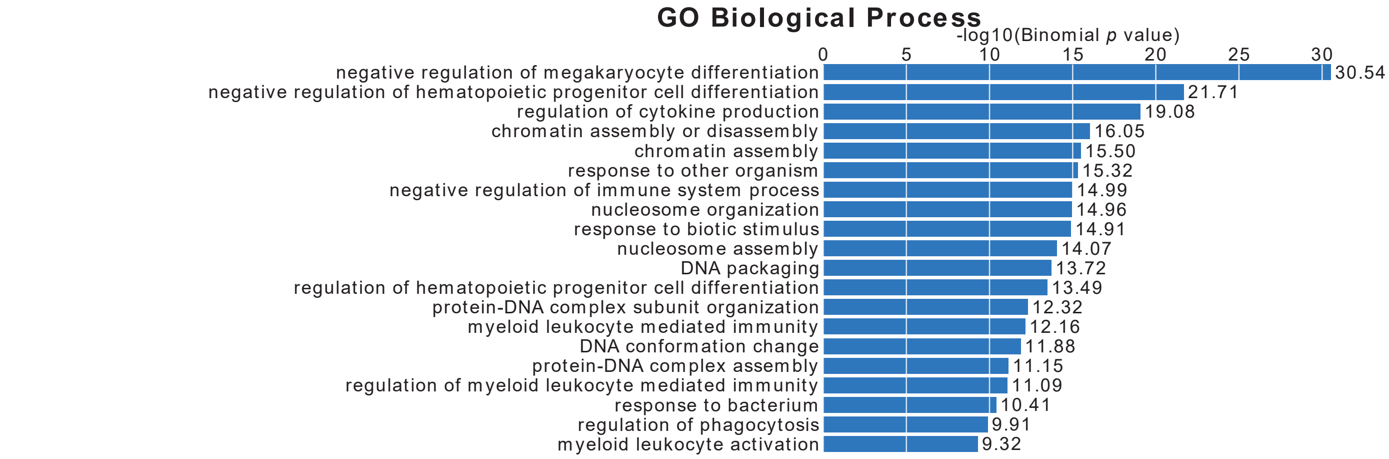

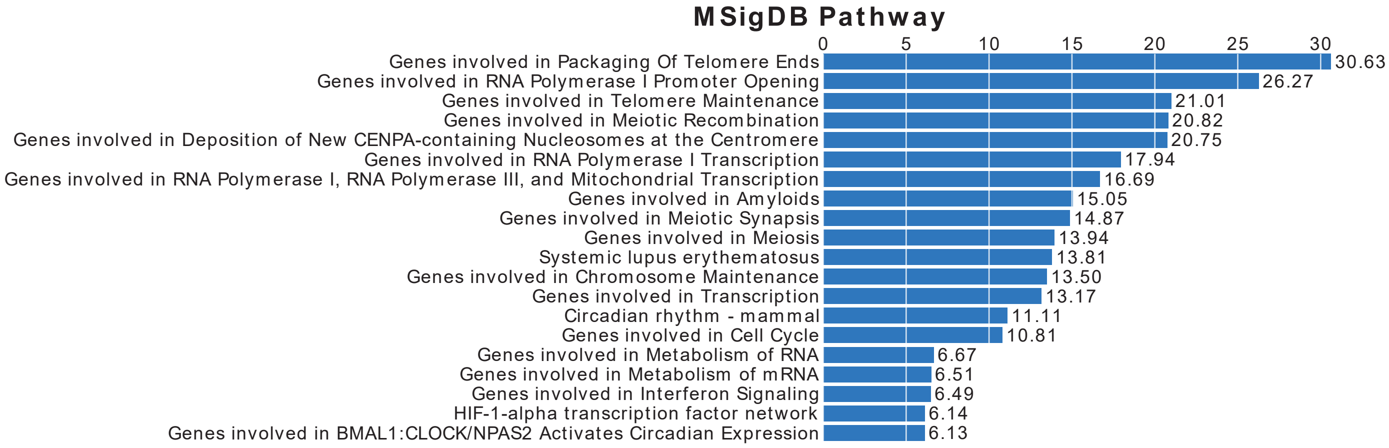

Liver

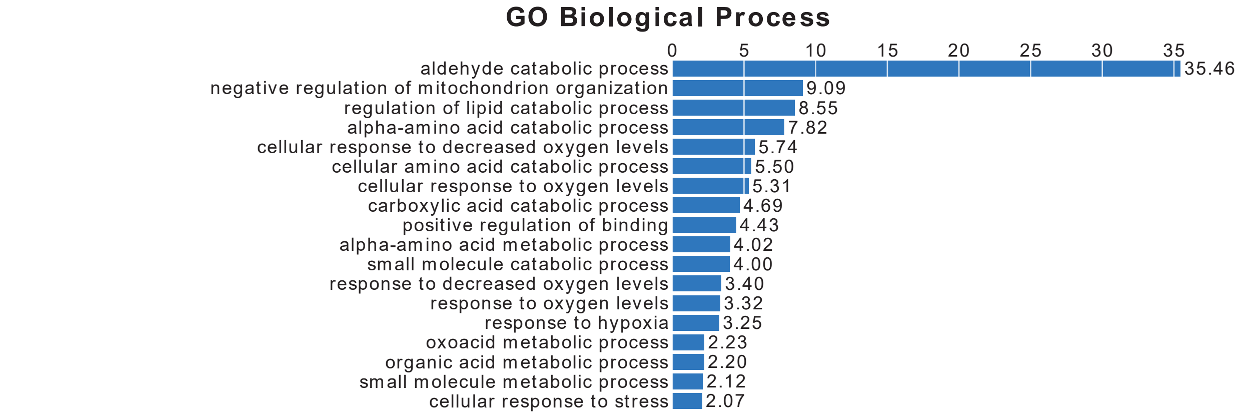

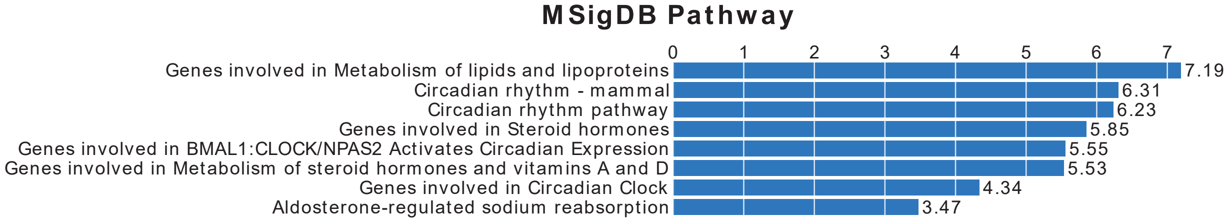

Oishi et al. Supplementary Figure S3

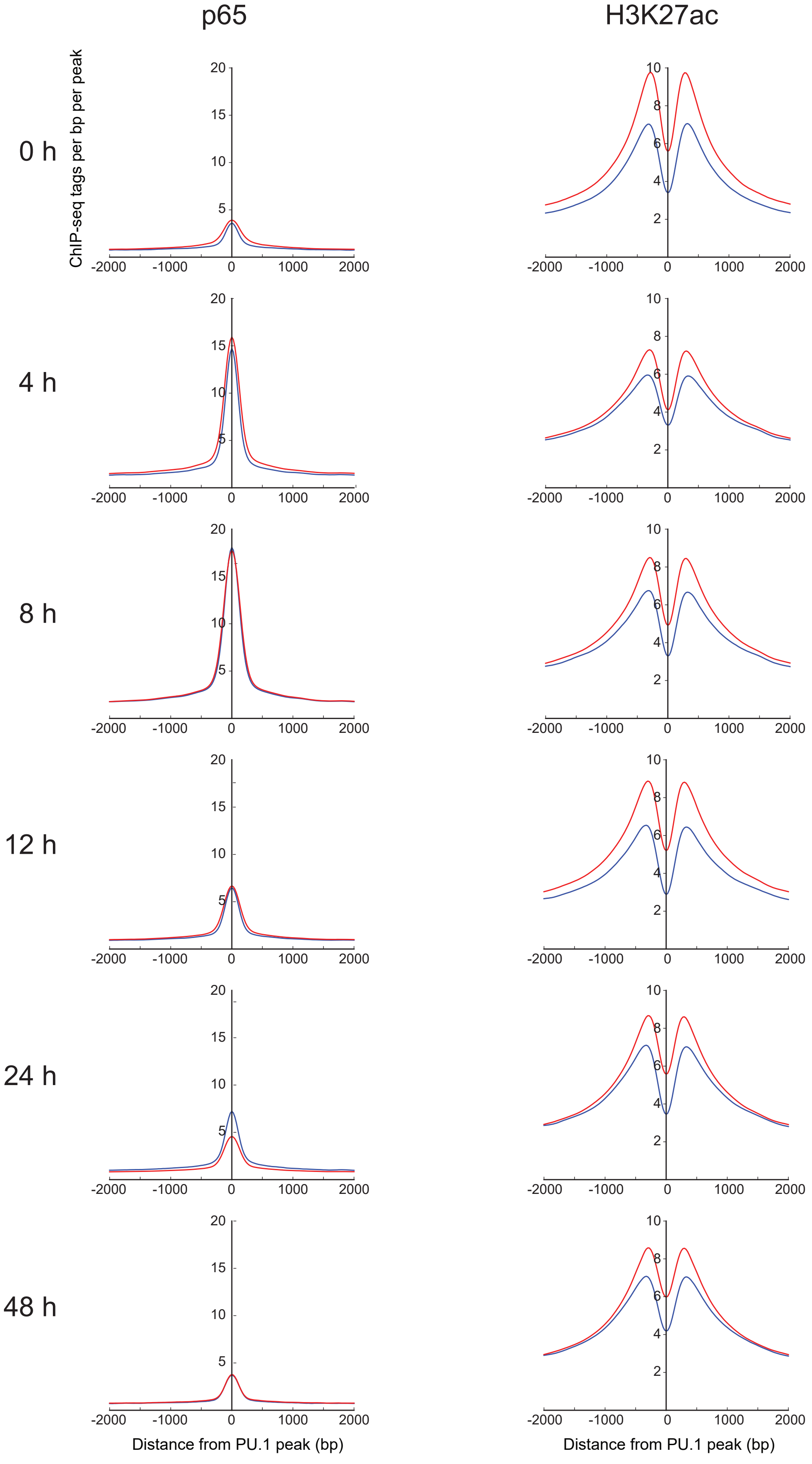

Oishi et al. Supplementary Figure S4
